# Modeling chronic infection with *Mycoplasma pneumoniae* at an air-liquid interface: global transcriptional response of HBEC3-KT respiratory epithelial cells to biofilm towers

**DOI:** 10.1128/iai.00137-26

**Published:** 2026-05-13

**Authors:** Rasha A. Fahim, Brynn G. Cole, Jessica E. Holland, Vanessa B. Polster, Mitchell F. Balish

**Affiliations:** 1 Department of Microbiology, Miami University 6403, Oxford, Ohio, USA; Tsinghua University, Beijing, China

**Keywords:** RNA-Seq, scanning electron microscopy, transepithelial electrical resistance, airway epithelium

## Abstract

*Mycoplasma pneumoniae*, a bacterial pathogen that causes chronic respiratory infections, grows as biofilm towers in both axenic culture and submerged tissue culture model systems. In these towers, virulence factor production is reduced. The clinical relevance of these towers is unclear because biofilms have not been examined in air-liquid interface (ALI) models of *M. pneumoniae* infection. We used differentiated HBEC3-KT human bronchial epithelial cells at an ALI to understand how *M. pneumoniae* grows on and interacts with airway cells in a more physiologically relevant setting than submerged systems, and to characterize the global transcriptional response of host cells in this context. We used scanning electron microscopy to examine the growth of *M. pneumoniae* on HBEC3-KT cells over time, employing a modified protocol to preserve mucus. This protocol revealed abundant biofilm towers associated with both ciliary tips and the mucus layer. RNA-seq analysis of HBEC3-KT cells at days 1 and 7 after infection indicated an early tempered cytokine response, followed later by induction of type III interferon, which is unexpected not only because that response is more typical of viruses, but also because *M. pneumoniae* is not known to enter host cells at an ALI. We assessed barrier function using transepithelial electrical resistance and culture of medium from the basal side of the host cells, revealing that disruption occurred, but only after prolonged infection. These results collectively suggest that *M. pneumoniae* limits damage to host cells when growing as biofilm towers by provoking only a selective inflammatory response, promoting chronic infection.

## INTRODUCTION

*Mycoplasma pneumoniae* is a common bacterial pathogen in the class Mollicutes that causes 20%–40% of all community-acquired pneumonia (CAP) cases (1, 2). CAP is a significant cause of hospitalization of both children and adults. A recent survey in the US found that 8% of hospitalized children diagnosed with CAP tested positive for *M. pneumoniae* (3). In China, 10%–30% of CAP cases in adults, teenagers, and children were found to be caused by *M. pneumoniae* (4, 5). The Centers for Disease Control and Prevention estimates that approximately 2 million people in the US are infected with *M. pneumoniae* yearly, and around 100,000 require hospitalization (6, 7). *M. pneumoniae* infection can also lead to other respiratory conditions like pharyngitis and tracheobronchitis and is associated with chronic conditions like asthma and reactive airway disease (2). The infection can also result in severe complications beyond the respiratory system (8). Thus, there is an urgent need for research to better understand and combat this pathogen. Given its significant clinical and public health implications, there is a need for ongoing research to better understand the pathogenesis, epidemiology, and antimicrobial resistance mechanisms of *M. pneumoniae* infection (9).

*M. pneumoniae* can form three-dimensional aggregates that, by analogy to other bacteria, have been termed biofilm towers, both without host cells and on submerged BEAS-2B bronchial cells (10–12). These structures exhibit high resistance to antibiotics and complement-mediated killing, consistent with chronic infection characteristics, but are susceptible to dual antibiotic treatment *in vitro* (12, 13). Although individual *M. pneumoniae* cells may play significant roles in disease, it is more likely that airway colonization by *M. pneumoniae* occurs from pre-formed aggregates than from individual cells (12). As *M. pneumoniae* aggregates develop into biofilm towers, there is a significant decrease in the production of virulence-related molecules, such as CARDS toxin, hydrogen sulfide, and hydrogen peroxide. This behavior may allow the bacteria to survive in a protected environment without triggering immune clearance, promoting chronic infections (11, 12).

Current *in vitro* models using submerged tissue culture have limitations due to the use of incompletely differentiated host cells (14). Although some studies have examined *M. pneumoniae* infection at an air-liquid interface (ALI), which better mimics *in vivo* conditions, these studies have not accounted for biofilm towers (15, 16). Culturing respiratory epithelial cells at an ALI allows them to develop into a more physiologically relevant, ciliated, pseudostratified epithelium with barrier function (17).

The occurrence and impact of biofilm towers formed by *M. pneumoniae* on well-differentiated respiratory epithelium remain unexamined, leaving a significant gap in our understanding of chronic *M. pneumoniae* infections. Prior research on *M. pneumoniae* in non-biofilm conditions demonstrated that the bacteria inflict considerable harm upon normal human bronchial epithelial (NHBE) cells, leading to cell death, decreased transepithelial electrical resistance (TEER), and migration of bacteria through the compromised epithelial layer (15). Considering that biofilm towers produce reduced amounts of virulence factors compared to individual bacterial cells, their impact on the respiratory epithelium at an ALI might be relatively modest (11, 12).

In this study, we sought to deepen our understanding of how interactions between *M. pneumoniae* and human airway cells evolve over time during persistent contact. To do this, we developed an airway infection model that mimics essential aspects of the host–pathogen interface. This ALI model supports the formation of a 3D-polarized epithelium. We selected HBEC3-KT, a human bronchial epithelial cell line commonly used in airway disease research that differentiates into a pseudostratified epithelium, forming cilia and producing mucus (17). We used microscopy to observe the growth and organization of *M. pneumoniae* during infection, analyzed the host response to infection using RNA sequencing, and assessed epithelial barrier integrity by measuring TEER. Our findings indicate that *M. pneumoniae*, in the form of biofilm towers, induces a temporally structured epithelial response, characterized by gene expression changes that promote early stress adaptation and barrier preservation, transitioning to a sustained, interferon-driven inflammation response and epithelial barrier weakening upon prolonged infection. Development of this model has provided a unique opportunity to gain deeper insight into the interactions between host and pathogen factors that influence disease progression, and will facilitate the evaluation of innovative therapeutic strategies under conditions that closely resemble clinical scenarios.

## RESULTS

### Distinct biofilm towers at ALI compared to submerged culture

**Fig 1:**
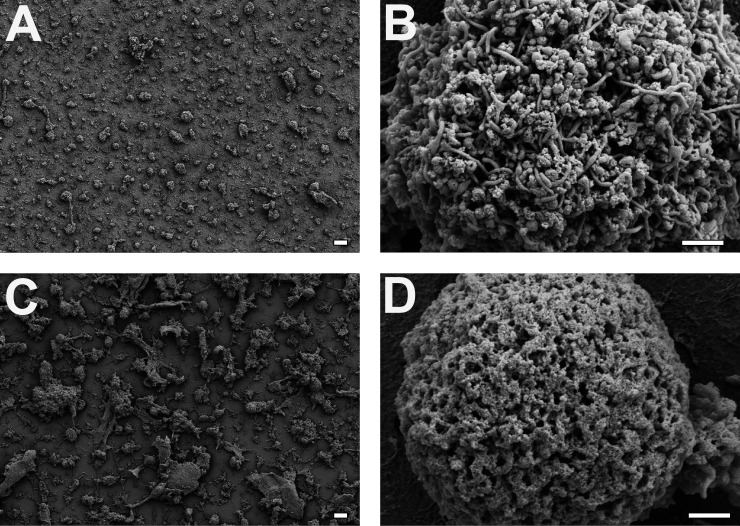
Scanning electron micrographs of *M. pneumoniae* biofilm towers grown on Transwell inserts. (**A and B**) *M. pneumoniae* biofilm towers grown submerged on the Transwell insert membrane at 4 days post-inoculation. (**C and D**) *M. pneumoniae* biofilm towers grown at ALI on the Transwell insert membrane at 4 days post-inoculation. Scale bar, 10 μm (A and C); 1 μm (B and D).

To investigate whether *M. pneumoniae* could form biofilm towers in an environment in which they were not covered with liquid media, mimicking the ALI of the respiratory tract, we inoculated *M. pneumoniae* directly onto Transwell insert membranes. After a few hours, the inoculum was removed to mimic the ALI conditions. Other Transwell inserts were inoculated with *M. pneumoniae* submerged in SP-4 medium remaining in the apical chamber, to allow visualization of differences between ALI and submerged conditions. When grown submerged, *M. pneumoniae* biofilm towers had an appearance comparable to biofilms grown on glass (11) and on BEAS-2B cells (12), with a mixture of rounded and elongated cells (Fig. 1A and B). When grown at ALI, biofilm towers still dominated the landscape, but they had a markedly different appearance (Fig. 1C and D). Few individual cells were attached to the substrate between the towers, unlike in submerged cultures. Additionally, the outer layer of the towers was characterized by a rough texture with many small nodules, possibly representing heavy coverage with extracellular polymeric substance, or alternatively as a result of desiccation due to air exposure.

### *M. pneumoniae* biofilm tower development on HBEC3-KT cells at ALI

**Fig 2:**
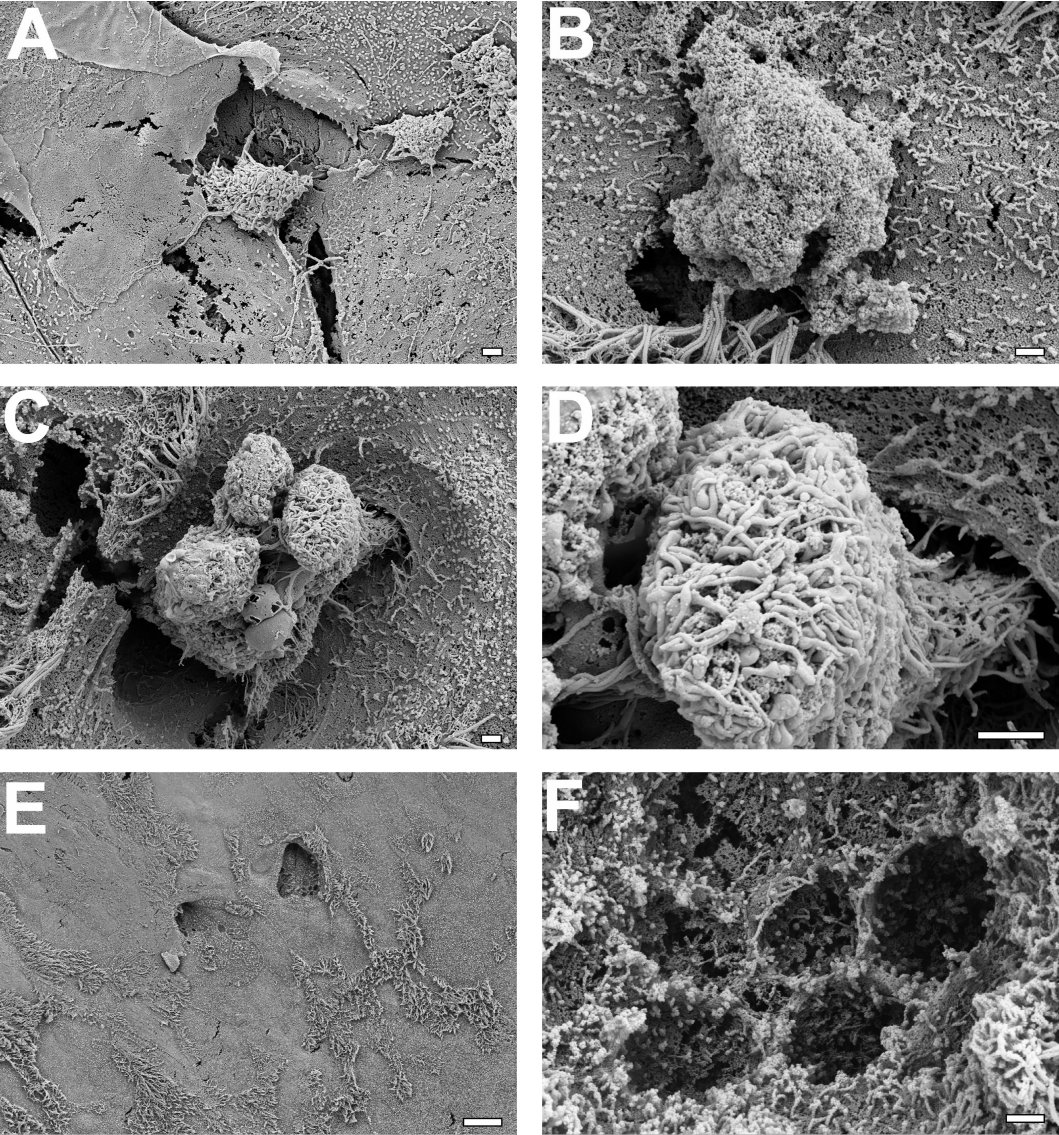
Scanning electron micrographs of *M. pneumoniae* biofilm towers on HBEC3-KT cells. *M. pneumoniae* biofilm tower structures at (**A**) D7 and (**B, C, and D**) D10. (**D**) Higher magnification shows rounded and elongated cells within the biofilm tower. (**E and F**) Holes in the epithelial monolayer of infected cells at D10. Scale bar, 1 μm (A–D and F); 20 μm (E).

SEM was used to observe the development of *M. pneumoniae* biofilm towers on infected HBEC3-KT cells at ALI at various time points. SEM images of uninfected HBEC3-KT cells showed an intact differentiated epithelial layer (Fig. S1A and B). By D5 (day 5 post-infection), when towers had grown and matured in axenic culture (18), very few small aggregates were visible anchored to HBEC3-KT cells (data not shown). By D7 (Fig. 2A), *M. pneumoniae* had begun to form small tower-like structures that were not as robust as those observed *in vitro* at the same time point. At D10 (Fig. 2B through D), biofilm towers on HBEC3-KT cells were distinctly larger. Importantly, the frequency with which we observed these structures was unexpectedly low, a matter to which we returned later (see below). Although some of the towers had the classical appearance of *M. pneumoniae* grown in submerged culture, with a mixture of features resembling both round and elongated bacteria (18; Fig. 2D), usually their appearance was very similar to biofilms grown at ALI in the absence of host cells (Fig. 1C, D and 2B). At sites where towers were present, the surfaces of infected epithelial cells generally featured distinct depressions or holes (Fig. 2E and F), reminiscent of what was observed in *M. pneumoniae*-infected normal human bronchial epithelial (NHBE) cells (15). At D10, multiple holes were observed in the epithelial layer, even in locations where biofilm towers were not present, which were never seen in the uninfected controls.

### Preservation of biofilm towers in mucus by LRR fixation

**Fig 3:**
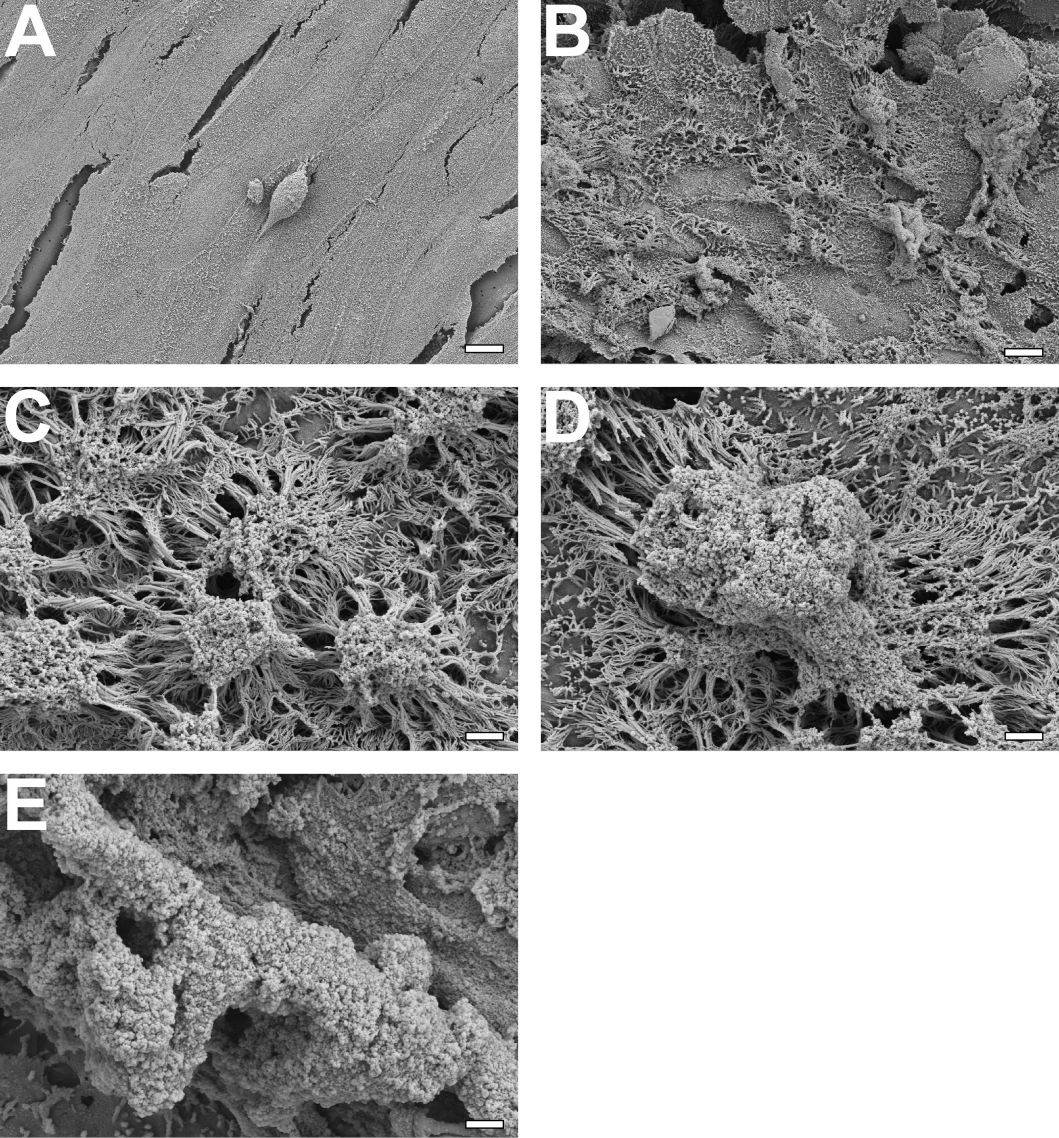
Scanning electron micrographs comparing standard fixation of *M. pneumoniae* biofilm towers on HBEC3-KT cells to LRR fixation at day 10 post-infection. (**A**) Standard fixation protocol; (**B–E**) LRR fixation protocol. Scale bar, 10 μm (A and B); 2 μm (C and D); 1 μm (E).

Because differentiated HBEC3-KT cells produce mucus at ALI (19), we speculated that the dearth of biofilm towers observed might be due to loss of mucus and other cell-derived material during sample preparation for SEM imaging. To address this, we applied a fixation protocol previously developed to partially preserve mucus (20, 21). Using this LRR fixation protocol, we visualized a much higher density of *M. pneumoniae* biofilm towers on the surfaces of infected cells (Fig. 3A and B) that closely resembled those seen in previous ALI experiments (see Fig. 1C and 2B). These towers, which varied in size but were often as small as ~2 µm in diameter, appeared on ciliary tips (Fig. 3C through E), consistent with a previous study that used different imaging techniques and cell lines (22). These towers often featured open spaces that likely serve as channels for nutrient access (Fig. 3E; see Fig. 2B).

### Global transcriptional changes in HBEC3-KT cells in response to *M. pneumoniae* infection

We aimed to analyze the host transcriptional response to infection as a means of focusing further research on the interplay between *M. pneumoniae* and airway epithelial cells. We evaluated the HBEC3-KT transcriptome at D1 and D7 to capture the early and late responses, respectively. The later time point may be more closely related to features of chronic infections.

**Fig 4:**
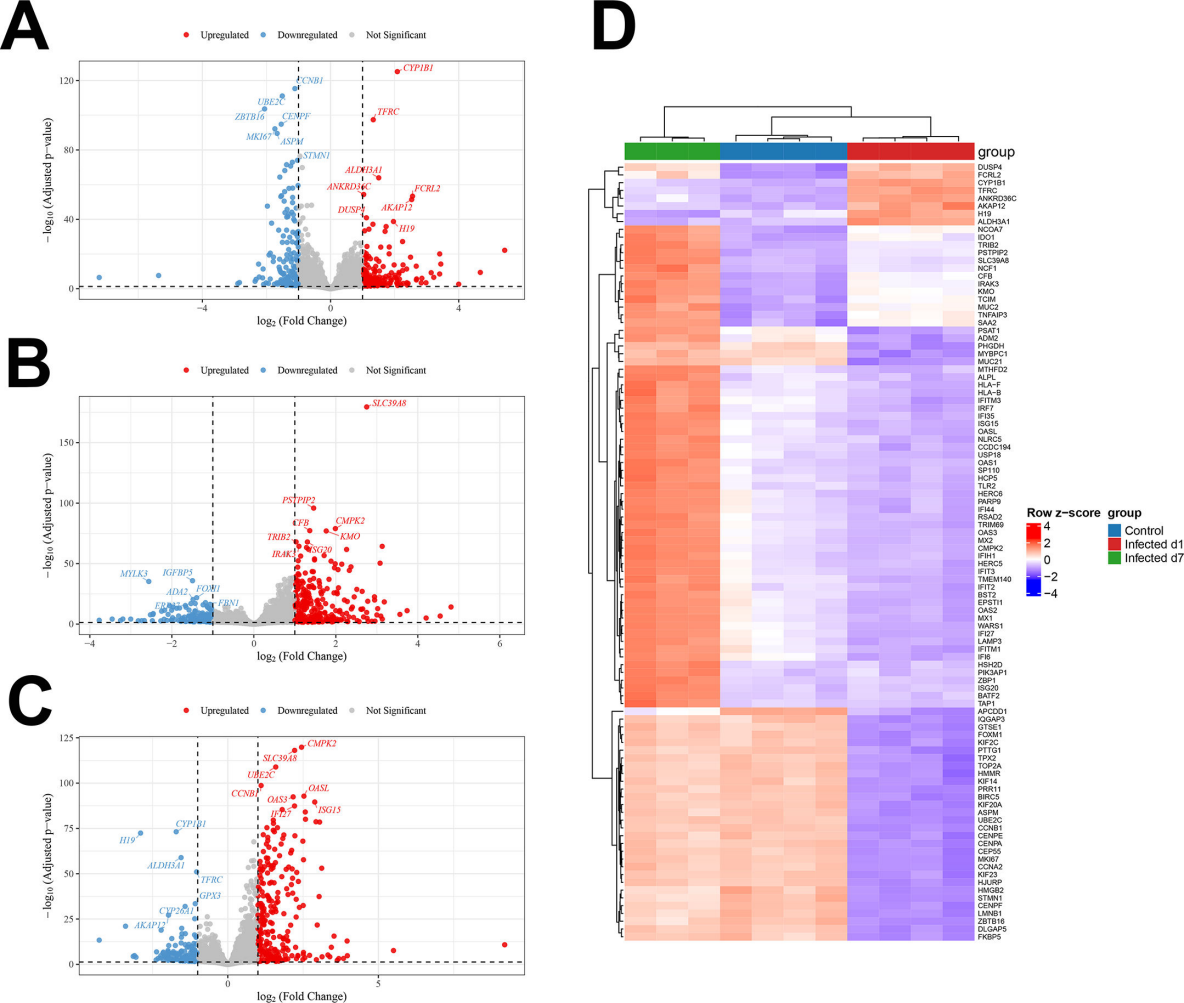
Global transcriptional changes in HBEC3-KT cells in response to *M. pneumoniae* infection. Volcano plots show the distribution of DEGs for each comparison: (**A**) D1 vs control; (**B**) D7 vs control; (**C**) D7 vs D1. The x-axis represents log₂ fold change, and the y-axis represents −log₁₀ adjusted *P*-value. DEGs were defined using thresholds of |log₂(fold change)| ≥ 1 and adjusted *P*-value (Benjamini–Hochberg) < 0.05. Red, upregulated genes; blue, downregulated genes; gray, non-significant genes. The genes with the most significant *P*-values are labeled. (**D**) Heat map and hierarchical clustering dendrogram of the top 100 DEGs (padj < 0.05 and |log₂(fold change)| > 1). VST expression values were z-score-normalized by gene and hierarchically clustered across samples. Colors represent relative expression across samples; higher z-scores (red) indicate expression above the gene’s mean, and lower z-scores (blue) indicate expression below the mean. Columns present control, D1, and D7, and each row represents an individual gene.

Analysis of gene expression in ALI cultures showed significant changes at both D1 and D7, compared with uninfected controls. There was substantial inter-group divergence and high intra-group similarity (Fig. S2). The genes that passed the threshold cutoff (adjusted *P* value [padj] < 0.05, |log₂FC| ≥ 1) were considered differentially expressed genes (DEGs). The analysis revealed 340 DEGs at D1 (184 upregulated and 156 downregulated), 411 DEGs at D7 (266 upregulated and 145 downregulated), and 416 DEGs for D7 vs D1 (261 upregulated and 155 downregulated) at D7 (Fig. 4A through C). A hierarchically clustered heat map of the top 100 DEGs (Fig. 4D) clearly showed groups of genes with highly consistent different expression change patterns among the three groups. The complete list of all DEGs is provided in File S1.

### Stress responses, cell cycle suppression, and limited inflammatory signaling in early infection

**Fig 5:**
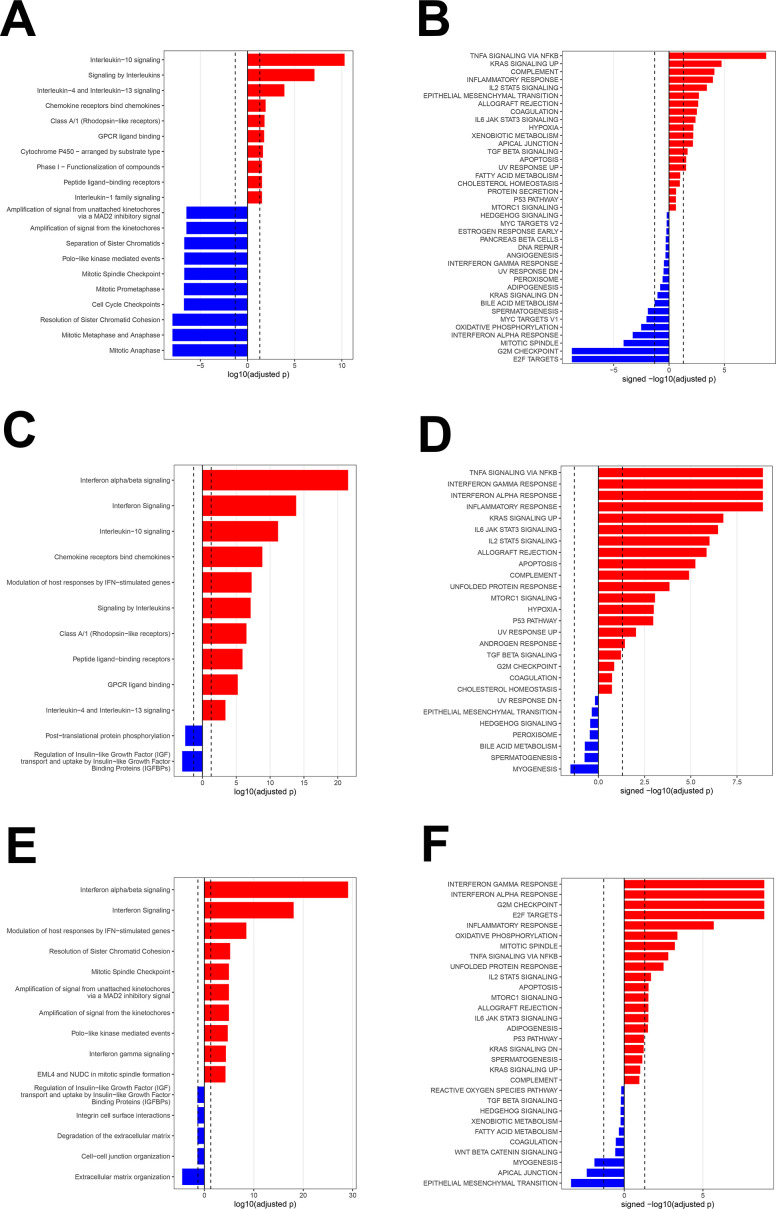
Pathway enrichment dynamics in response to *Mycoplasma pneumoniae* infection over time. (**A, C, and E**) Reactome pathway ORA; (**B, D, and F**) gene set enrichment analysis (GSEA). (**A and B**) D1 vs control; (**C and D**) D7 vs control; (**E and F**) D7 vs D1. Reactome ORA was performed using DEGs, whereas GSEA was performed using the full ranked gene list, as described in Materials and Methods. For Reactome panels, bars represent the top enriched pathways, shown with signed −log₁₀ (adjusted *P*-value), with positive (red) and negative (blue) values indicating enrichment among up- or downregulated genes, respectively. For GSEA panels, bars represent normalized enrichment scores summarized as signed −log₁₀(FDR), with dashed lines denoting the significance threshold (FDR = 0.05). Pathways were ranked by adjusted *P*-value. Up to the 20 most highly up- and downregulated pathways are shown for each comparison.

Four enrichment analyses were conducted to characterize the host response to infection and to identify the primary biological functions of the DEGs. Overrepresentation analyses (ORAs) (Reactome, KEGG, and GO) were performed for the DEGs, whereas pathway analysis was conducted using gene set enrichment analysis (GSEA) on all expressed genes, without applying a DEG cutoff. At D1, all analyses of biological pathways showed activation of cytokine, chemokine, and stress-related signaling pathways, whereas cell cycle processes and mitotic programs were significantly suppressed. Reactome analysis (Fig. 5A) demonstrated significant down-regulation of pathways related to the cell cycle. Conversely, most upregulated pathways involved cytokine and chemokine signaling, including IL-1, IL-4, IL-10, and IL-13 family pathways. Additionally, the increased expression of cytochrome P450 genes suggested early activation of detoxification and oxidative stress responses. GSEA enrichment analysis (Fig. 5B) further supported the activation of pro-inflammatory programs, whereas pathways associated with proliferation were significantly downregulated.

**Fig 6:**
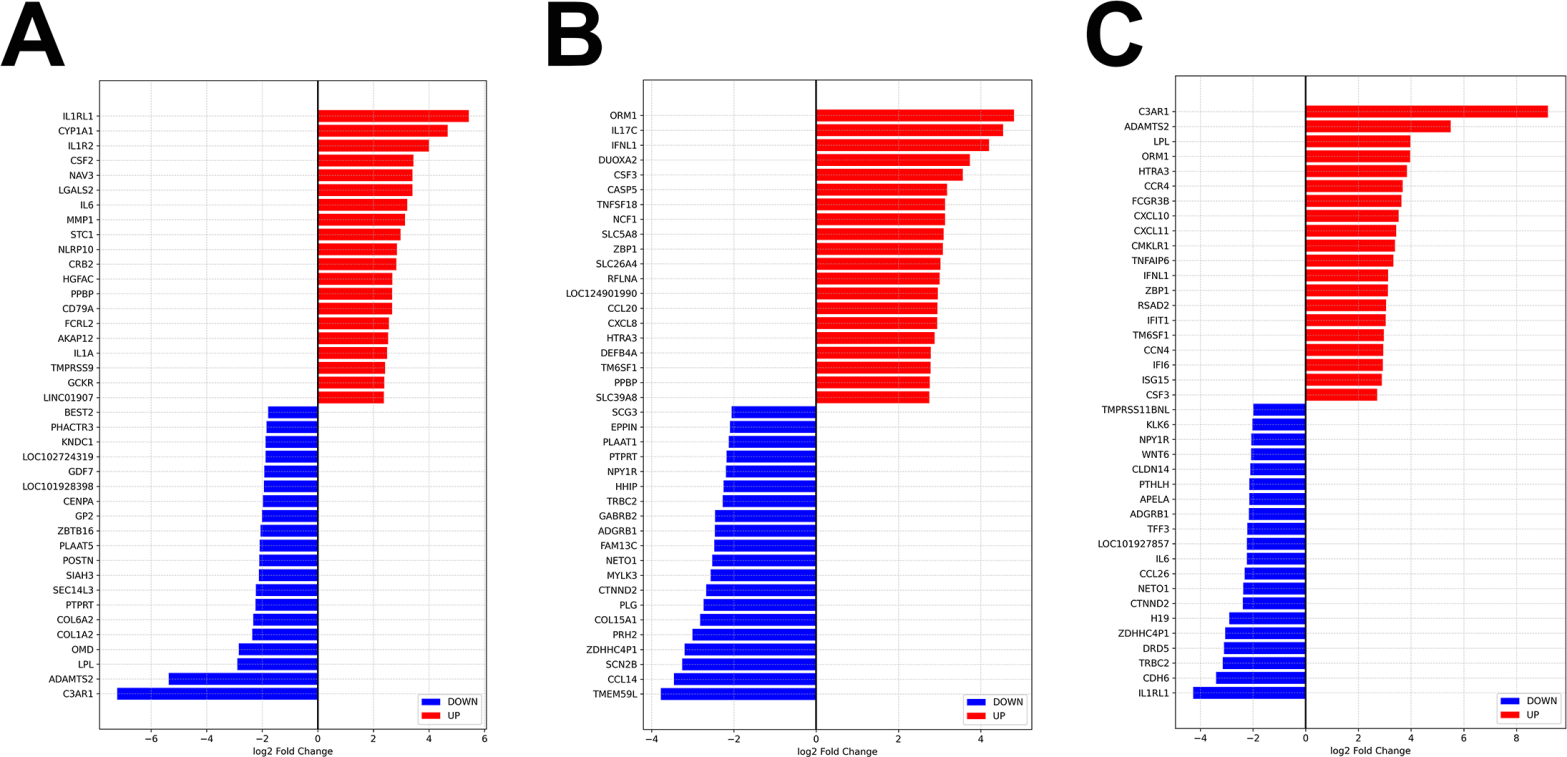
Top 20 up- and downregulated DEGs over the course of infection. Bar plot of DEGs that passed a threshold of adjusted *P*-value < 0.05 and |log₂fold change| > 1. (**A**) D1 vs control; (**B**) D7 vs control; (**C**) D7 vs D1. Upregulated (red) and downregulated (blue) genes ranked by absolute log₂ fold change. The x-axis represents log₂ fold change; the vertical line at zero indicates no change.

We then considered individual genes. At D1 (Fig. 6A), there was significant activation of oxidative stress and xenobiotic response genes (CYP1A1, STC1). There was increased MMP1 expression, but suppression of collagen and matrix synthesis genes (COL1A2, COL6A2, ADAMTS2, OMD). Although some pro-inflammatory pathways were upregulated at D1, there were also changes in expression of individual genes that suggested counteraction of inflammation. Genes encoding classical inflammatory cytokines (IL-1α, IL-6) were only modestly induced, and the upregulation of IL1R2, a decoy receptor, suggested inhibition of IL-1 signaling. The complement-associated gene C3AR1 and the Th2-related remodeling marker POSTN were downregulated. Several genes related to secretory functions and goblet cell activity (BEST2, GP2, SEC14L3) were also downregulated. Conversely, genes involved in maintaining cell polarity (AKAP12 and CRB2) were upregulated.

### Interferon signaling and broad inflammatory pathway activation late in infection

At D7, compared with uninfected control, Reactome analysis revealed an increase in the expression of pathways involving interferon-stimulated genes and cytokine-driven inflammatory signals (Fig. 5C). Significant downregulation was restricted to a limited number of pathways. GSEA confirmed widespread activation of pathways including interferon-alpha and -gamma responses, TNF-α signaling via NF-κB, IL-6/JAK/STAT3, IL-2/STAT5, and complement (Fig. 5D). These results imply that by D7, the epithelial cells were producing an interferon-driven response and an altered cytokine response in comparison to D1.

By D7, at the individual gene level (Fig. 6B; File S1), the epithelial transcriptome also showed a notable shift toward increased inflammatory and antimicrobial activity. There was a strong induction of genes encoding classic neutrophil-recruiting chemokines (CXCL8, CSF3; 23) and IL-17C, indicating activation of mucosal inflammatory pathways. Multiple genes associated with interferon signaling (IFNL1, ZBP1, CASP5) (24–26) were upregulated. There was also a broad activation of interferon-stimulated genes (ISGs), including multiple OAS family members, MX1/MX2, ISG15, IFIT genes, RSAD2, and interferon regulatory factors (25, 27). Taken together, these results indicate a coordinated interferon-associated transcriptional program. Genes involved in structural and homeostatic functions, including those for ECM components, lipid and surfactant metabolism, and adherence regulation (COL15A1, CTNND2, PLAAT1, PTPRT), were down-regulated, suggesting weakening of epithelial integrity.

### Changes in airway epithelial transcriptional response over the course of *M. pneumoniae* infection

To evaluate how the epithelial response evolved during infection, we directly compared gene expression profiles on D7 with those on D1. Both Reactome and GSEA analysis identified interferon signaling pathways as being significantly increased at D7 compared with D1 (Fig. 5E and F). Additionally, there was widespread suppression of epithelial structure, adhesion, ECM organization, and metabolic pathways. KEGG and GO enrichment analyses supported these findings (Fig. S3).

Expression of the genes encoding IL-6 and IL-23α was distinctly reduced at D7 compared with D1 (Fig. S4). In contrast, some cytokine genes, including TNF and IL1B, remained elevated. Interestingly, the genes encoding CXCL1 and CXCL10, which have pro-inflammatory activity through the recruitment of other immune cells, were upregulated at D7, likely in response to increased TNF and interferon, respectively. From D1 to D7, the host response shifted from a mild IL-1–stress and detoxification profile to a significantly more intense, interferon-driven inflammatory state (Fig. 6C). Multiple cell cycle and mitotic genes that had been suppressed at D1 showed increased expression at D7. In parallel, several genes associated with epithelial secretory programs, including BEST2, GP2, SCG3, and TFF3, were significantly downregulated from D1 to D7. Several mucin genes exhibited differential expression during infection, with distinct temporal patterns (Fig. S5). On D1, MUC5AC and MUC2 were significantly upregulated, whereas by D7, MUC2 and MUC13 showed sustained or increased expression, while MUC5AC and MUC16 expression were decreased.

**Fig 7:**
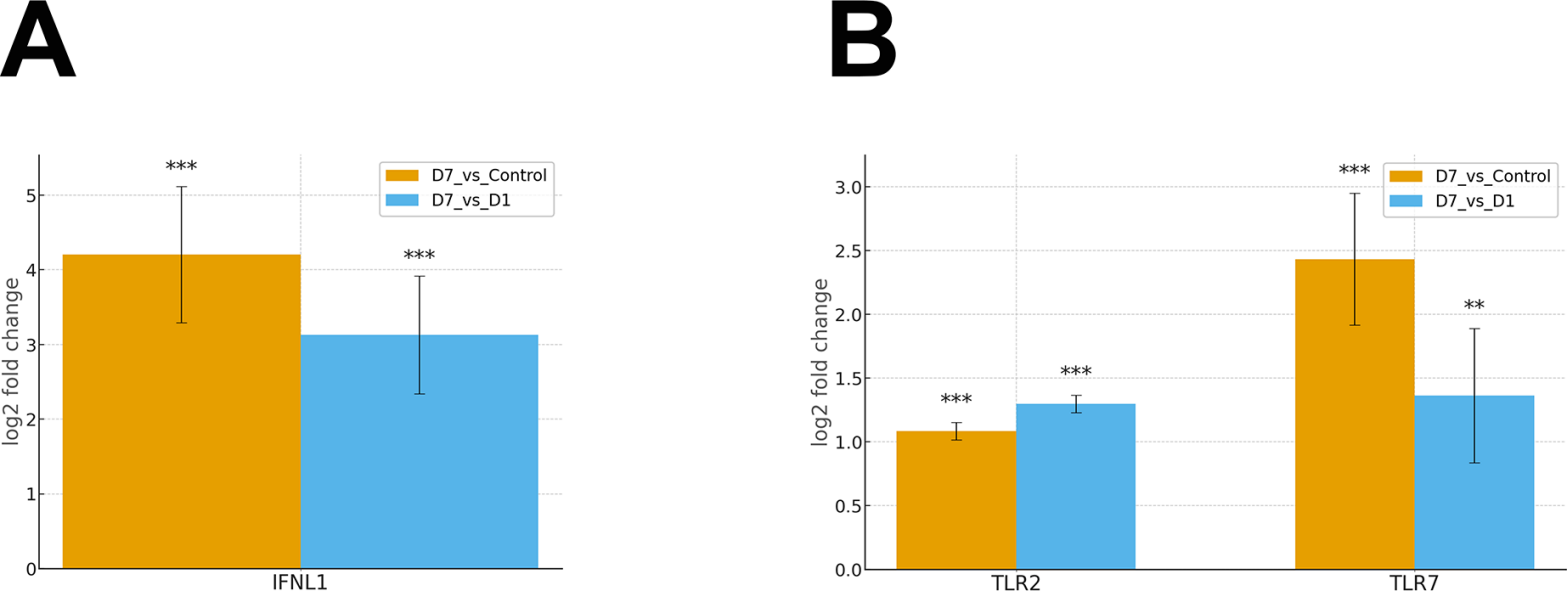
Differential expression of TLR and interferon-related genes. Bar plots show log₂ fold-change values for (**A**) IFNL1 and (**B**) TLR2 and TLR7 at D7 vs control or D1. Asterisks denote significance (*padj < 0.05, **padj < 0.01, ***padj < 0.001). Bars represent DESeq2-estimated log2 fold changes for the indicated contrasts; error bars indicate the standard error of the estimated log2 fold change (lfcSE).

Interestingly, on D7 (Fig. 7A and B), IFNL1 expression was significantly higher than in uninfected controls and further elevated compared with D1, indicating progressive induction of type III interferon signaling. Additionally, expression of the pattern recognition receptors TLR2 and TLR7 was significantly higher on D7 than in the control and D1 groups, aligning with the stronger activation of innate immune sensing pathways observed at the later stage of infection.

### Epithelial barrier function during the course of infection

**Fig 8:**
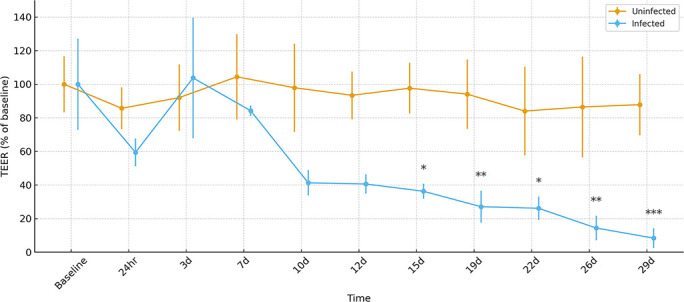
Epithelial barrier function during infection. Transepithelial electrical resistance (TEER) was measured in infected and uninfected cells at various time points up to D29. TEER values were normalized to each group’s baseline (set to 100%) for visualization purposes. Data are presented as mean ± SD from three biological replicates per condition at each time point. A one-way ANOVA with Tukey’s HSD post hoc test was used to assess statistical significance between infected and uninfected groups at each time point. (*P < 0.05, **P < 0.01, ***P < 0.001).

The early increase in expression of genes like AKAP12 and CRB2 (Fig. 6A) suggested that the barrier function of the epithelium would be preserved early in infection. However, both the observation of holes in the barrier at later time points and the down-regulation of TFF3, CDH6, and tight junction protein-coding genes at D7 (Fig. 6C; File S1) suggested potential weakening of the barrier that could lead to dissemination of *M. pneumoniae*. We tested whether *M. pneumoniae* had breached the barrier formed by HBEC3-KT cells by plating medium from the basal chamber on SP-4 agar plates at various time points after infection. We did not detect a quantifiable amount of *M. pneumoniae* in the basal medium until D10, after which there was a substantial increase over time (Table 1). We also directly quantified barrier integrity by measuring TEER at various time points. Although TEER began to drop notably after D7, we did not detect a statistically significant decrease in TEER in infected cells until D15 (Fig. 8). The decrease continued over time, suggesting continued loss of barrier integrity.

**TABLE 1:** *M. pneumoniae* recovered from basal chamber

Days post infection	CFU/mL
10	2,400
12	4,000
15	6,500
19	8,400
22	13,800

## DISCUSSION

*In vitro* and submerged tissue culture models for *M. pneumoniae* biofilm towers have been fruitful, but respiratory tract epithelial cells grown at an ALI, with development of their apical surfaces, mucin production, and barrier function, better model the environment encountered by airway pathogens (15, 28). Understanding how *M. pneumoniae* biofilm towers develop and impact epithelial cells at an ALI is the next logical step toward understanding chronic *M. pneumoniae* disease.

Following infection of differentiated HBEC3-KT cells, *M. pneumoniae* formed biofilm tower-like structures that were anchored to the epithelium, rather than individual bacteria (Fig. 2). Likewise, very few individual bacteria were present when *M. pneumoniae* was grown axenically at an ALI (Fig. 1). These results suggest that biofilm towers might actually be the dominant form of *M. pneumoniae* in an airway infection, emphasizing the significance of studies of biofilms in this organism. The initial impact of these towers was relatively modest, consistent with reduction in virulence factor production *in vitro* (11). However, at later time points, this interaction damaged the integrity of the epithelial monolayer, occasionally creating perforations (Fig. 2 and 8). A previous study of *M. pneumoniae* infection of a differentiated bronchial epithelium (15) revealed the development of furrows in the monolayer late in infection, and these furrows contained a high density of *M. pneumoniae* by immunofluorescence microscopy. This study was not performed in the context of biofilms, but our results suggest that the furrows correspond to the holes we describe, and the high density of *M. pneumoniae* corresponds to biofilm towers such as those we have observed.

SEM images prepared with standard fixation agents showed only bare epithelial cells or scattered bacteria at far lower density than expected (Fig. 3A). We suspected that this apparent loss of material was attributable to failure to preserve mucus. We therefore turned to the LRR protocol for sample preparation because it improves preservation of mucus by stabilizing negatively charged moieties, acidic polysaccharides, and lipids (20, 21, 29). As a result, we saw a dramatic increase in the abundance of mycoplasmas in our SEM images (Fig. 3B). Although biofilms in direct contact with the airway mucosa are expected to be most relevant to disease processes, they are rarely observed *in situ* (30). The term “biofilm” frequently appears in cystic fibrosis (CF) and chronic obstructive pulmonary disease (COPD) literature, but it refers to bacteria grown from sputum or bronchoalveolar lavage samples, rather than to directly observed biofilms (27). Our data support the idea that the limited detection of mucosa-associated biofilms in CF and COPD may reflect methodological and clinical sampling constraints rather than their actual absence (31). Airway biopsies often exclude the overlying mucus layer, where bacterial aggregates may preferentially reside (32, 33). In addition, clinical biopsy processing involves tissue rinsing and fixation protocols that disrupt mucus structure and associated microbial communities (31, 34). Moreover, previous patient treatments can complicate detection because mucolytic therapies change mucus viscosity and structure, and antibiotics can decrease bacterial biomass (35–37).

Transcriptomic analysis showed that in response to *M. pneumoniae* infection, epithelial cells activated stress response pathways and repressed cell cycle and mitotic processes, possibly as a protective strategy to prevent pathogens from hijacking proliferative processes and to prioritize cell survival. Early in infection, there was increased expression of genes whose products are involved in detoxifying harmful substances and managing oxidative stress, such as cytochrome P450 enzymes (38) (Fig. 5). These responses likely help epithelial cells neutralize bacterial metabolites and mitigate oxidative damage caused by the infection. Genes involved in modulating the composition of the extracellular matrix and the expression of tight junction components underwent changes in expression on both D1 and D7 (Fig. 6). We tested whether there were structural changes weakening the epithelial barrier later in infection, and observed a late decline in TEER and an increase in bacterial translocation (Fig. 8; Table 1), validating this prediction. Prince et al. (15) also reported late paracellular migration of *M. pneumoniae* in an NHBE model. The regulation of mucin gene expression after infection illustrates the dynamic nature of the epithelium. Initially, the expression of gel-forming mucin genes, such as MUC5AC and MUC2, increased (39) (Fig. S5), likely creating a protective barrier to trap and eliminate pathogens. However, as infection progressed, selective suppression of certain mucins occurred alongside sustained expression of others, suggesting a restructuring of the mucosal barrier possibly related to the observed loss of barrier integrity.

A mixed inflammatory and anti-inflammatory response occurred at the transcriptional level following *M. pneumoniae* infection of HBEC3-KT cells. At D1, although some cytokines and chemokines were activated, the overall inflammatory response was limited (Fig. 5), in agreement with what was previously described for cytokine production in response to *M. pneumoniae* in primary human bronchial epithelial cells (16). For instance, the expression of classical pro-inflammatory cytokine genes like IL1A and IL6 was only slightly increased, whereas expression of the IL-1 decoy receptor IL1R2 suggested active dampening of inflammatory signaling (40, 41) (Fig. 6). Cells at D7 exhibited a series of coordinated changes in cytokine gene expression involving both pro- and anti-inflammatory responses. Together with the reduction in virulence factor production in *M. pneumoniae* biofilms (11), it appears that the strategy of this organism is to carry out limited damage, perhaps toward a goal of maintaining a hospitable environment during infection.

The response at D7 was dominated by a significant induction of type III interferon signaling, with expression of both IFNL1 and ISGs increasing (Fig. 6 and 7). The simultaneous increase in TLR2 and TLR7 expression further indicates an enhanced innate immune response as the infection advances (42) (Fig. 7). TLR7 and IFNL1 were induced only when the epithelial barrier was beginning to break down. Activation of an interferon response is typically a response to viruses or intracellular bacteria (43, 44), but *M. pneumoniae* has been characterized as extracellular in another ALI model (15). However, type III interferons can also be induced by TLRs located on the plasma membrane that recognize various extracellular molecules (45). The induction of IFNL1 at D7 could therefore be a response to some product or component of *M. pneumoniae*, even if it is exclusively extracellular. Interestingly, host extracellular microRNAs, like miR-34a, which is induced upon infection with *M. pneumoniae*, serve as TLR7 ligands (46, 47).

Collectively, our data support a model in which *M. pneumoniae* establishes a prolonged interaction with the airway epithelium in the form of biofilm towers, with low virulence factor production, contacting both the cell surfaces and the mucus. Initially, this interaction triggers a stress adaptation response and controlled immune signaling, but over time, it shifts toward interferon-driven and inflammatory reactions, which, together with remodeling of the extracellular matrix and tight junctions, leads to epithelial dysfunction and barrier disruption. This progressive weakening of epithelial integrity provides a mechanistic framework for understanding how *M. pneumoniae* infections may persist and contribute to chronic airway disease, as well as cause extrapulmonary complications through dissemination to distant sites. Further work aimed at identifying the *M. pneumoniae*-derived molecules that enable them to form biofilms and elicit critical changes in the host cells will be important for generating new and effective therapeutic targets to reduce chronic infection. Additionally, therapeutic strategies targeting epithelial repair pathways or modulating interferon signaling may help reduce long-term damage in chronic *M. pneumoniae* infections.

## MATERIALS AND METHODS

### Bacterial strains and culture conditions

*M. pneumoniae* strain M129 was grown in tissue culture flasks with 10 mL of SP-4 broth (48) and incubated at 37°C until reaching mid-to-late log phase, when the medium had just turned yellow. For infection experiments, frozen stocks were resuspended in SP-4 broth and incubated at 37°C for at least 2 h before use.

### HBEC3-KT cell passaging, expansion phase, and cryopreservation

HBEC3-KT cells (CRL-4051, ATCC; 17) were cultured following the vendor’s instructions. After thawing, its contents were transferred to a centrifuge tube containing 3 mL of complete PneumaCult Ex Medium (5008, Stemcell Technologies), with supplements included as per the manufacturer’s recommendations, and mixed gently. Then, 7 mL of additional complete medium was slowly added. The mixture was centrifuged at 350 × *g* for 5 min at room temperature. The pellet was resuspended in 3 mL of fresh medium. Cells were stained with trypan blue, and viable cells were counted with a hemocytometer. In a 25-cm² culture flask, 2.5 × 10^5^ cells (1 × 10^4^ cells/cm^2^) were seeded in 5 mL of complete PneumaCult Ex medium and incubated at 37°C and 5% CO_₂_. Medium was changed every 2–3 days until reaching confluency, approximately 5–7 days.

### HBEC3-KT cell expansion in Transwell inserts followed by differentiation at ALI

After expanding in tissue culture flasks, the cells were further expanded in Transwell inserts (CLS3470, Corning). Briefly, the cells were washed with 5 mL of D-PBS (without Ca²^+^ and Mg²^+^) (37,350, Stemcell Technologies), then 2 mL of 0.025% trypsin-EDTA (25200-056, Gibco) in D-PBS was added. The flask was incubated at 37°C for 5–7 min to detach the cells. After trypsin neutralization, the cells were centrifuged and resuspended in 2 mL of complete PneumaCult-Ex medium as described above. A viable cell count was performed as described above. Then, 500 μL of complete expansion medium was added to the basal chamber of the Transwell insert, and 200 μL to the apical chamber. Next, 3.3 × 10⁴ cells were seeded in the apical chamber and incubated at 37°C under 5% CO_₂_. The medium was changed every 2–3 days until full confluency was reached, approximately 2–4 days. At 100% confluency, the medium was removed from the apical chamber to begin differentiation. Then, 500 μL of complete PneumaCult-ALI medium (5001, Stemcell Technologies), with supplements included as per the manufacturer’s instructions, was added to the basal chamber and changed every 2-3 days until the cells were fully differentiated, approximately 3-4 weeks. Mucus was produced as expected (17), and excess mucus was washed away at least once a week starting in the second week by washing the apical chamber with 200 μL of D-PBS. Additionally, TEER values confirmed the development of epithelial barrier integrity, rising from 50 to 80 Ω·cm^2^ in undifferentiated cells to 900–1,200 Ω·cm^2^ in differentiated cells.

### *M. pneumoniae* grown axenically on Transwell insert membranes

*M. pneumoniae* cultures were prepared as described above. To mimic the ALI conditions of the respiratory epithelium, *M. pneumoniae* was inoculated onto the apical surface of the Transwell insert without any host cells, with SP-4 medium in the basal compartment. After a few hours, the inoculum was removed to maintain the ALI. For submerged conditions, *M. pneumoniae* was added to the apical surface of the membrane while including medium both basally and apically.

### HBEC3-KT cell infection

Fully differentiated HBEC3-KT cells in the apical compartment of the Transwell insert were washed with 200 μL of D-PBS and then infected with *M. pneumoniae* in SP-4 prepared as described above at an MOI of 500 (12). The cells were then incubated at 37°C under 5% CO_₂_ for 4 h. Afterwards, the bacterial inoculum was removed, and the cells were incubated for various time points up to 29 days. Uninfected differentiated HBEC3-KT cells and SP-4-treated differentiated cells served as controls. Medium in the basal compartment was changed every 2–3 days.

### *M. pneumoniae* biofilm tower visualization by scanning electron microscopy

For the standard fixation protocol, HBEC3-KT cells were washed with D-PBS and fixed on the insert membrane with 1.5% glutaraldehyde/1% formaldehyde/0.1 M sodium cacodylate, pH 7.2, for 30 min at room temperature. Inserts were rinsed with sodium cacodylate for 30 min and dehydrated using an ethanol series (25%–100%, vol/vol) followed by treatment with hexamethyldisilazane (HMDS). The membrane was cut, attached to aluminum stubs, and gold-coated by sputtering. Samples were analyzed with a Zeiss Supra 35 FEG-VP scanning electron microscope at the Miami University Center for Advanced Microscopy and Imaging. To preserve mucus, the lysine acetate-ruthenium red (LRR) fixation protocol was used as previously described (20, 21). Then, dehydration, drying, coating, and visualization were performed as described above. At least four independent visualization experiments were performed per time point. Axenically grown *M. pneumoniae* biofilm towers were visualized using LRR fixation.

### RNA extraction, sequencing, and transcriptome analysis

HBEC3-KT cells infected with *M. pneumoniae*, as well as uninfected cells, were scraped directly from the Transwell membrane into DNA/RNA Shield (R1100-50, Zymo Research). The lysate was transferred to a microcentrifuge tube and stored at −80°C. RNA extraction and sequencing were performed at the Rush University Genomics Core (Chicago, IL, USA). RNA was purified using the Promega Maxwell RSC simplyRNA Cells Kit (AS1390). RNA quality was evaluated using High Sensitivity RNA ScreenTape Analysis (5067-5579 and 5067-5580, Agilent) and RNA ScreenTape Analysis (5067-5576 and 5067-5577). Polyadenylated transcripts were enriched with NEXTFLEX Poly(A) Beads 2.0 (NOVA-512995, PerkinElmer/NovaBio), followed by directional library construction using the NEXTFLEX Rapid Directional RNA-Seq Automation Kit 2.0 (NOVA-5198-53). Poly(A) selection and library preparation were carried out on the Revvity Sciclone G3 NGSx iQ Workstation. Final libraries were sequenced on an Illumina platform. Raw FASTQ files were processed via standard RNA-seq workflow. Initial read quality was assessed with FastQC (49). Adaptor sequences and low-quality bases were trimmed with fastp (50). Trimmed reads were aligned to human genome assembly GRCh38 using STAR with default parameters for paired-end RNA-seq (51). STAR output, sorted BAM files, and alignment metrics supported downstream QC. MultiQC compiled quality reports, confirming consistent sequencing and mapping (File S2; 52). Principal component analysis (PCA) for variance-stabilizing transformed (VST) values revealed outliers via PC1 z-scores (±2.5) and Mahalanobis distance (*P* < 0.05). One sample (D7C) above thresholds across the top 2,000–10,000 variable genes was removed due to deviation. Others remained within acceptable ranges (53, 54). This sample was excluded from further analysis (Fig. S6).

### Comparison of host transcriptional profile

Gene-level read counts were generated using featureCounts, including only uniquely aligned reads. The count matrix served as input for differential expression analysis in R. DESeq2 estimated size factors and dispersions through the DESeq() pipeline (55), with Wald tests comparing day 1 (D1) vs. control, day 7 (D7) vs. control, and D7 vs. D1. DEGs were defined as padj <0.05, and |log₂ (fold change)|> 1 applied to volcano plots and DEG counts (56). PCA of VST-transformed data and a heatmap of the top 100 DEGs visualized group clustering (55, 57). DEGs were used for ORA to identify enriched pathways among DEGs using GO, KEGG, and Reactome databases. Separate analyses were performed for up- and down-regulated genes (58–60). Significance was tested with a hypergeometric test, and Benjamini–Hochberg correction was applied; pathways with false discovery rate (FDR) < 0.05 were considered enriched. Enriched pathways were ranked by FDR for visualization (60, 61). Signed bar plots illustrated pathways: positive for up-regulated and negative for down-regulated genes, with bar length indicating −log₁₀(FDR). Dashed lines marked the FDR = 0.05 threshold. Gene Set Enrichment Analysis (GSEA) was performed on all genes tested by DESeq2 without applying a differential expression cutoff. Genes were ranked based on log₂ fold change for each comparison (57, 62). GSEA utilized the MSigDB Hallmark gene set. Significant pathways were identified using Normalized enrichment scores (NES) and adjusted *P*-values, with an FDR < 0.05 indicating significance (63). Positive NES indicated upregulated genes, while negative NES indicated downregulated genes (62). Results were presented with signed bar plots as described above. The bar plots were generated using ggplot2 in R.

### *M. pneumoniae* detection in the basal compartment

Medium from the basal compartment was collected at various time points to test for the presence of *M. pneumoniae*. Then, 50 μL of the media was plated on SP-4 agar plates and incubated at 37°C for 5–7 days, and colonies were counted to determine the CFU/mL. These experiments were performed in duplicate.

### Transepithelial electrical resistance measurement (TEER)

The EVOM Manual Meter (EVM-MT-03-02, World Precision Instruments) was used to measure TEER per manufacturer instructions. Prior to each set of measurements, the electrode was sterilized in 70% isopropyl alcohol for 15 min and rinsed with D-PBS. At each time point, medium was removed from the basal compartment. The apical compartment was rinsed with D-PBS to remove mucus. Next, 500 μL of D-PBS was added to the basal chamber, and 200 μL to the apical chamber. The long electrode was placed in the basal chamber, and the short in the apical chamber. The blank insert contained no epithelial cells. Resistance was calculated by subtracting the blank value, and TEER (Ω·cm²) was obtained by multiplying resistance by 0.33. Experiments were performed in biological triplicate using separate samples for each time point. Significance was determined by one-way ANOVA + Tukey HSD with *P* < 0.05. Data are mean ± SD, normalized to baseline.

## Data Availability

Raw FASTQ files have been submitted to the NCBI SRA database and are available under accession number PRJNA1427893.

## SUPPLEMENTAL MATERIAL

Data S1 iai.00137-26-s0001.xlsx Differentially expressed genes.

Data S2 iai.00137-26-s0002.txt RNA-Seq QC data.

Fig. S1 iai.00137-26-s0003.tif SEM images of uninfected cells.

Fig. S2 iai.00137-26-s0004.tif PCA plot for transcriptomics samples.

Fig. S3 iai.00137-26-s0005.tif Gene ontology/KEGG pathways.

Fig. S4 iai.00137-26-s0006.tif Chemokine/cytokine changes.

Fig. S5 iai.00137-26-s0007.tif Differential expression of mucin genes.

Fig. S6 iai.00137-26-s0008.tif Outlier detection analysis.

Supplemental material iai.00137-26-s0009.docx Legends for Data S1 and S2.

ASM does not own the copyrights to Supplemental Material that may be linked to, or accessed through, an article. The authors have granted ASM a non-exclusive, world-wide license to publish the Supplemental Material files. Please contact the corresponding author directly for reuse.
