# Supplementary figures and images for: Modeling chronic infection with Mycoplasma pneumoniae at an air-liquid interface: global transcriptional response of HBEC3-KT respiratory epithelial cells to biofilm towers
Source: Infect Immun. 2026 May 13;94(6):e00137-26. doi: 10.1128/iai.00137-26 (PMC13248681; doi:10.1128/iai.00137-26)

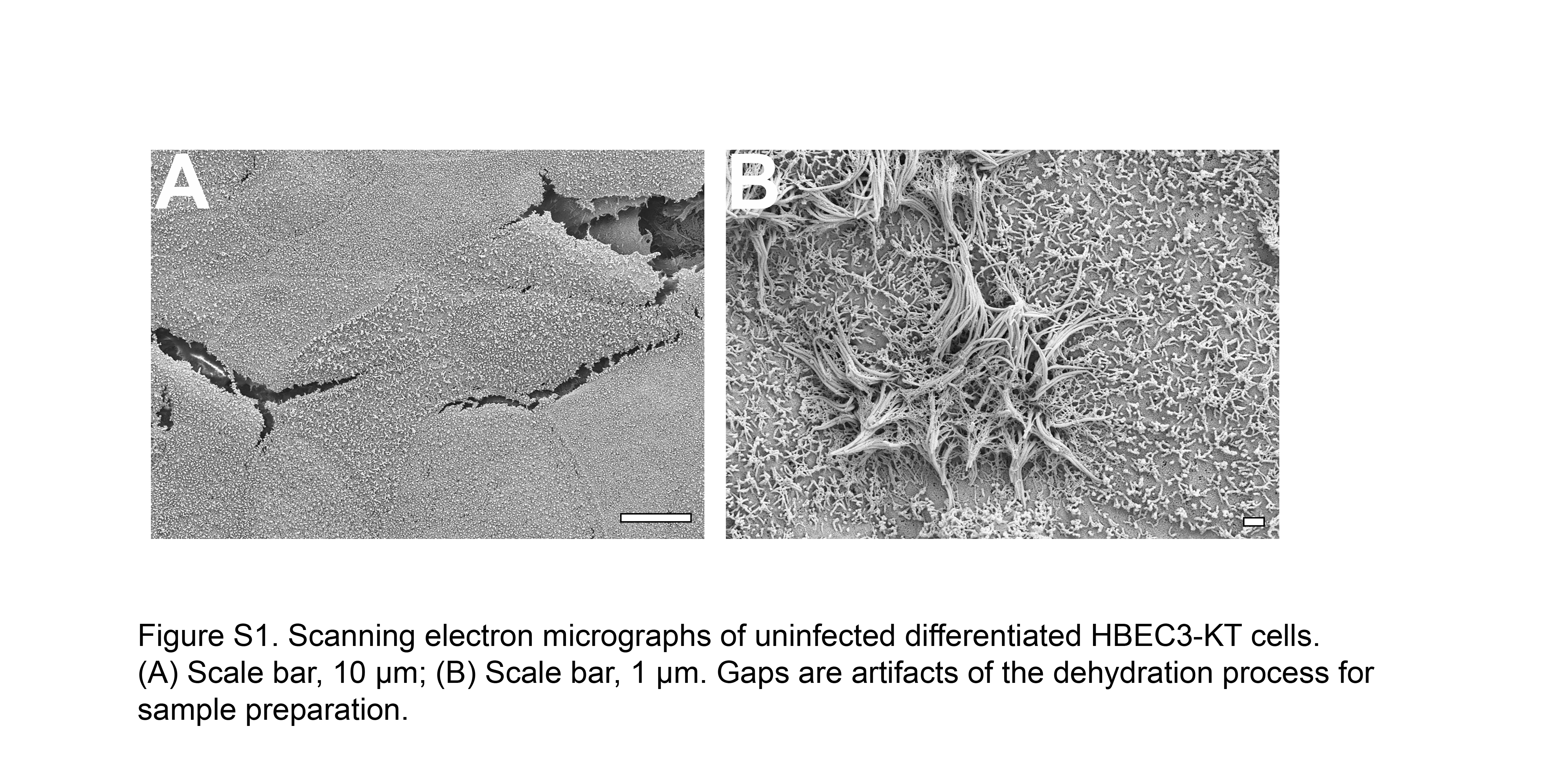

Supplement: Fig. S1 — SEM images of uninfected cells. [file iai.00137-26-s0003.tif]

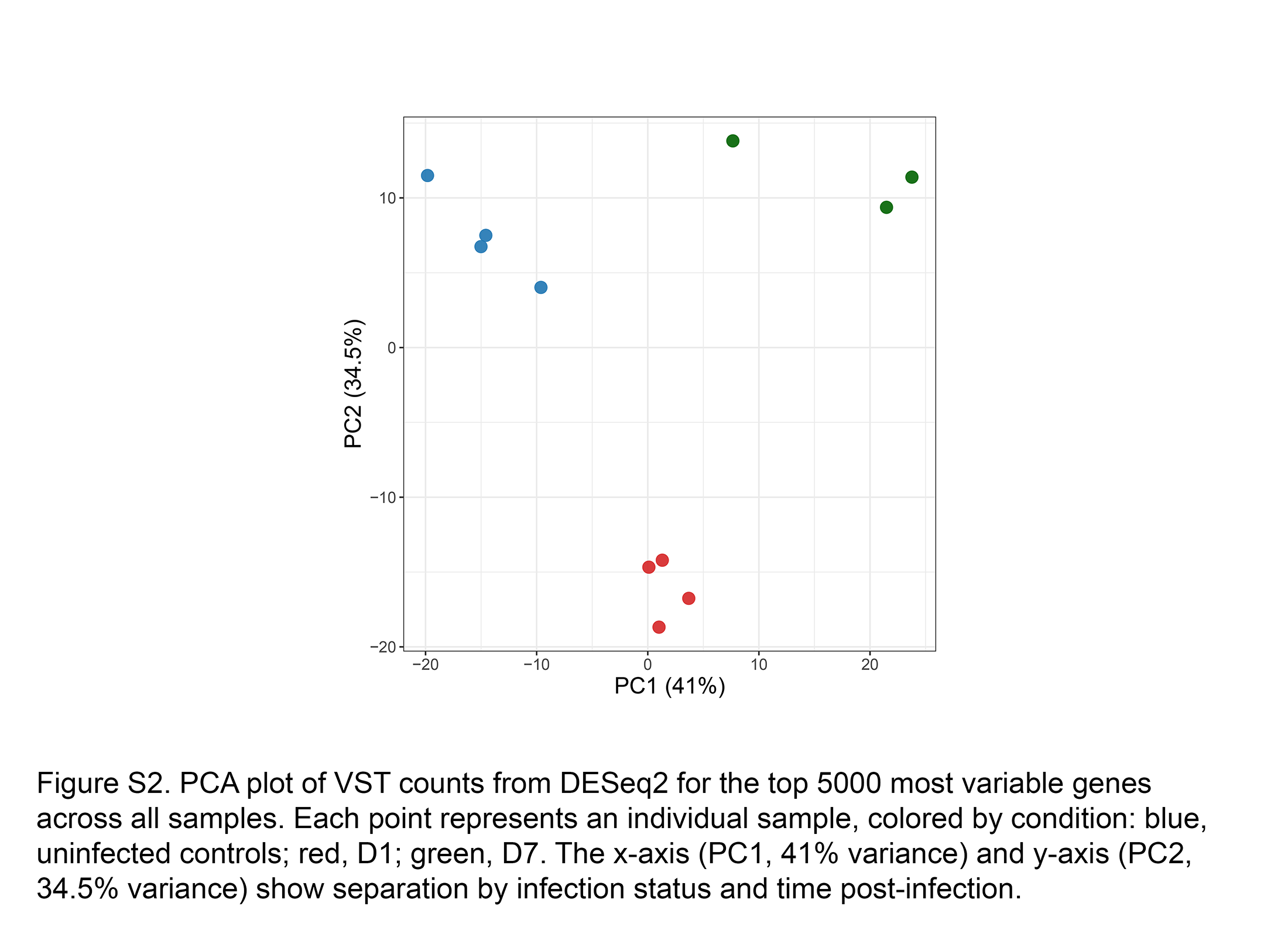

Supplement: Fig. S2 — PCA plot for transcriptomics samples. [file iai.00137-26-s0004.tif]

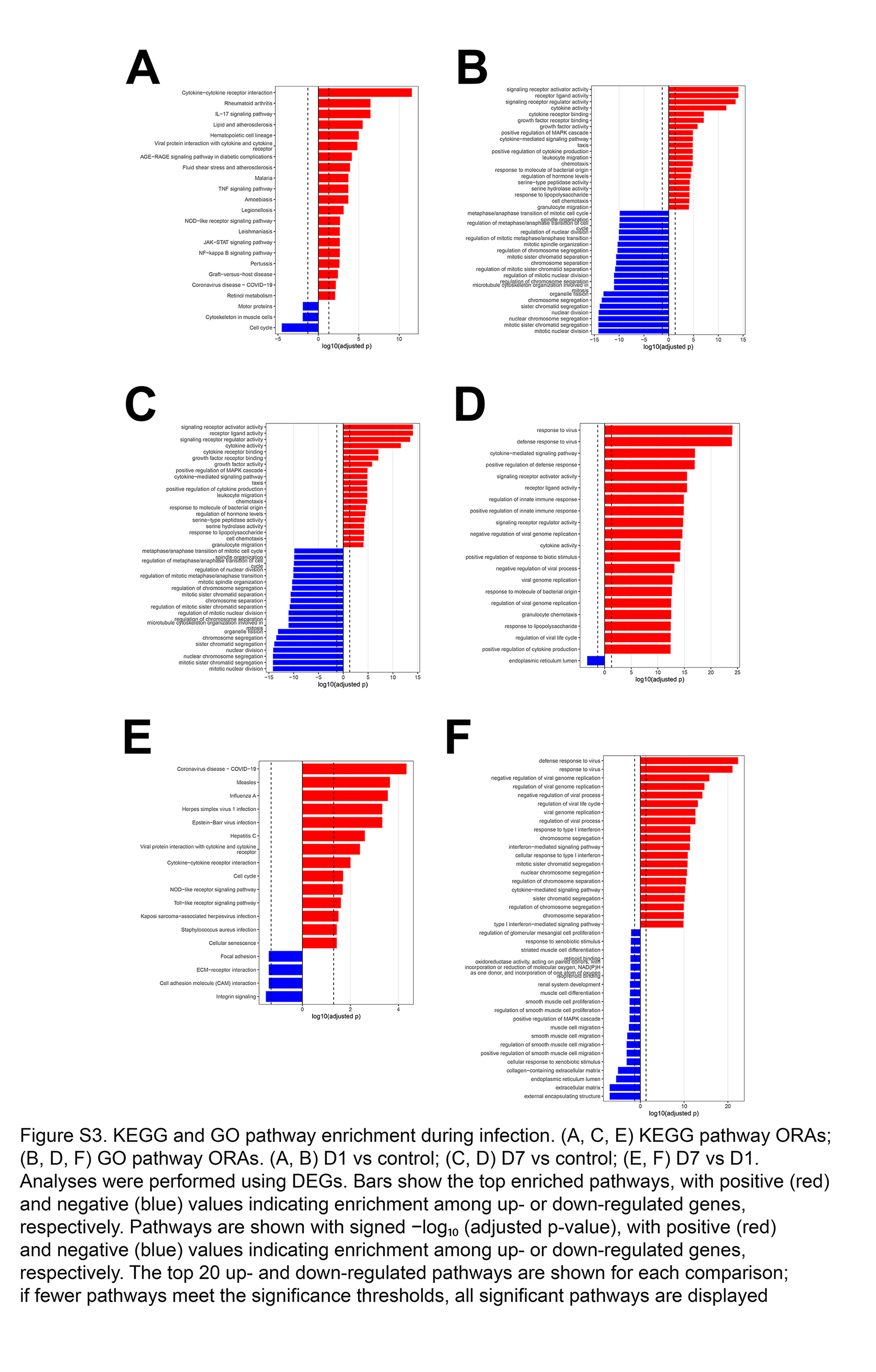

Supplement: Fig. S3 — Gene ontology/KEGG pathways. [file iai.00137-26-s0005.tif]

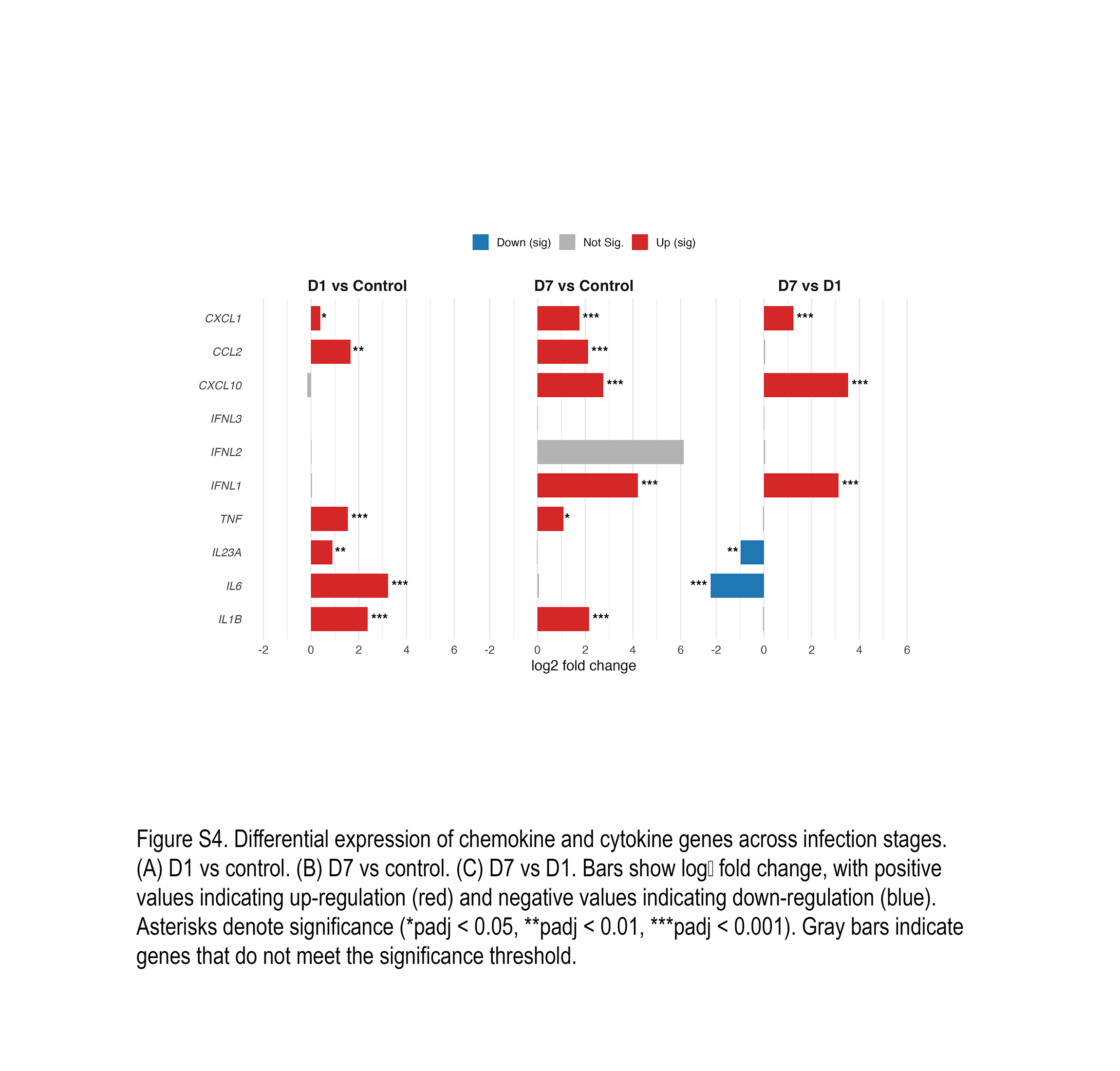

Supplement: Fig. S4 — Chemokine/cytokine changes. [file iai.00137-26-s0006.tif]

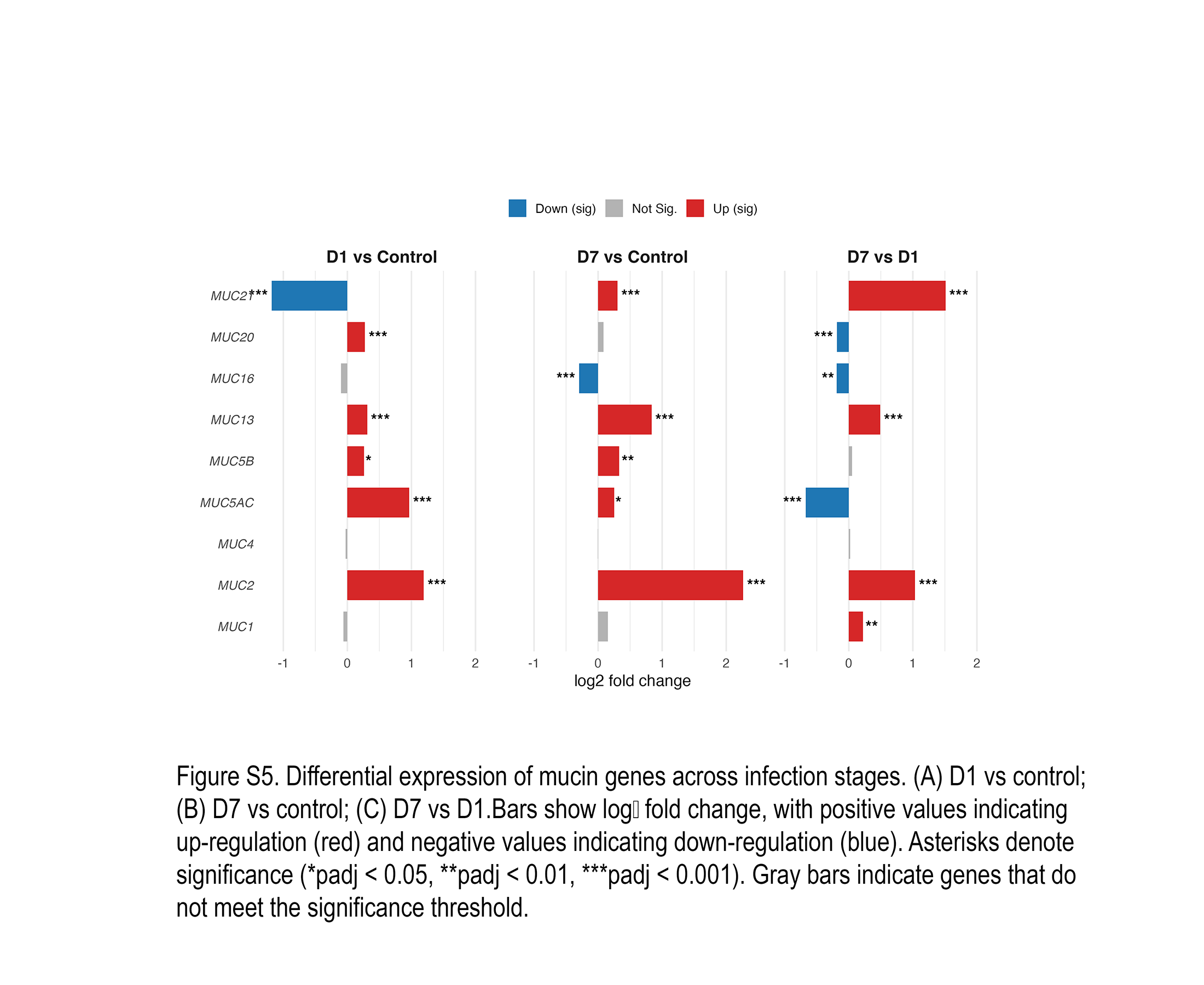

Supplement: Fig. S5 — Differential expression of mucin genes. [file iai.00137-26-s0007.tif]

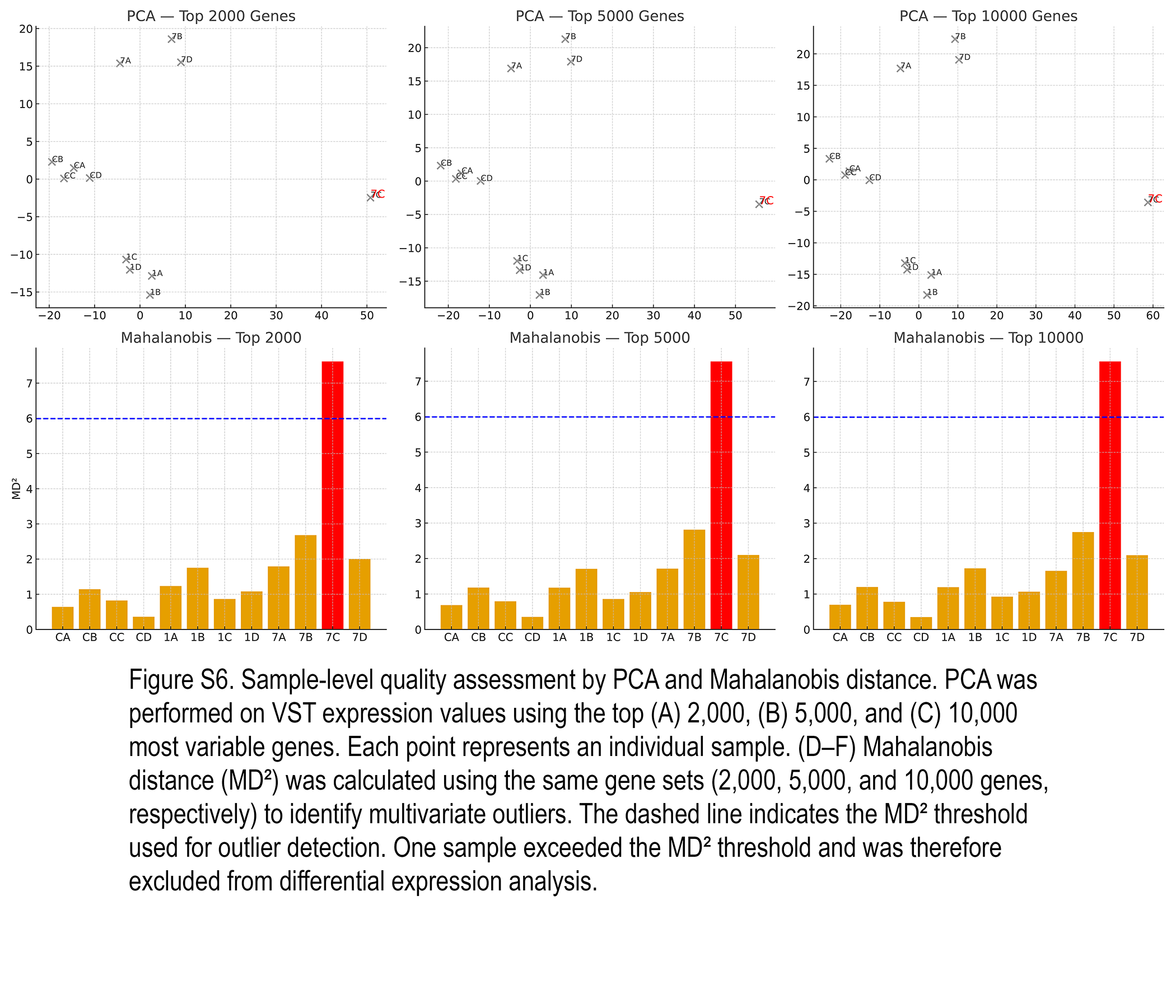

Supplement: Fig. S6 — Outlier detection analysis. [file iai.00137-26-s0008.tif]
