# Supplementary material for: Modeling chronic infection with Mycoplasma pneumoniae at an air-liquid interface: global transcriptional response of HBEC3-KT respiratory epithelial cells to biofilm towers
Source: Infect Immun. 2026 May 13;94(6):e00137-26. doi: 10.1128/iai.00137-26 (PMC13248681; doi:10.1128/iai.00137-26)
Supplement: Supplemental material — Legends for Data S1 and S2. [file iai.00137-26-s0009.docx]

Supplementary File S1

Differentially expressed gene (DEG) data. Statistical data for all HBEC3-KT cell genes that meet the threshold for differential expression, as described in Materials and Methods, are included, both alphabetically for all genes and in order of p value for the top 100 DEGs.

Supplementary File S2

MultiQC report for RNA-Seq samples showing alignment percentage for each sample, as described in Materials and Methods.
